# Reversed Potts Shunt and Venopulmonary Extracorporeal Membrane Oxygenation Support in Sustained Pulmonary Hypertension Crisis

**DOI:** 10.1016/j.jaccas.2025.105153

**Published:** 2025-09-24

**Authors:** Xiafeng Yu, Yanjun Sun, Lijun Fu, Zhuoming Xu, Yumin Zhong, Wenjing Hong, Yi Yan, Yinyu Yang, Hao Zhang

**Affiliations:** a Department of Cardiothoracic Surgery, Shanghai Children's Medical Center, Shanghai Jiao Tong University School of Medicine, Shanghai, China; b Department of Cardiology, Shanghai Children's Medical Center, Shanghai Jiao Tong University School of Medicine, Shanghai, China; c Department of Radiology, Shanghai Children's Medical Center, Shanghai Jiao Tong University School of Medicine, Shanghai, China

**Keywords:** pulmonary hypertension crises, reversed Potts shunt, venopulmonary ECMO

## Abstract

**Background:**

Infantile idiopathic pulmonary arterial hypertension complicated by recurrent episodes of pulmonary hypertensive crisis is an extremely rare and critical condition with high mortality.

**Case Summary:**

We present the case of a 5-month-old infant with a pulmonary hypertension crisis refractory to medical therapy, who underwent successful treatment with an emergency reversed Potts shunt (RPS), along with postoperative venopulmonary extracorporeal membrane oxygenation (V-P ECMO) support.

**Discussion:**

From an imaging perspective, the mechanism by which RPS improves symptoms in patient with sustained pulmonary hypertension crises is explained. Additionally, this is the first reported case of successful postoperative use of V-P ECMO support after RPS.

**Take-Home Messages:**

It is important to recognize the importance of RPS and V-P ECMO in the treatment of pulmonary hypertension crises. By comparing pre- and postoperative imaging data, the mechanism by which RPS improves cardiac function is better understood.

## Graphical Abstract

**Figure:**
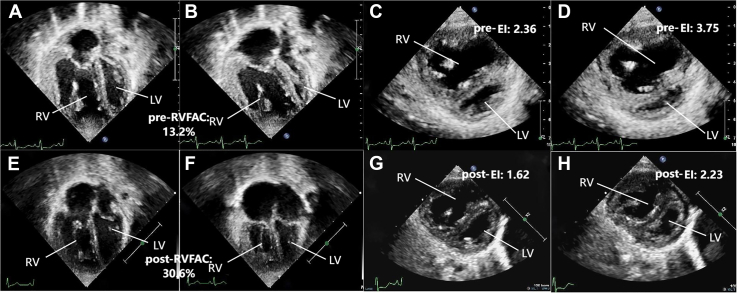

## History of Presentation

A 5-month-old boy, weighing 6.6 kg, with idiopathic pulmonary arterial hypertension presented with gradually worsening cyanosis of the lips after crying over the past one-half month, received oral bosentan (12 mg twice a day) and sildenafil (3 mg twice a day). The patient was admitted for cyanosis exacerbation and syncope after crying spell 1 day prior. Echocardiogram demonstrated mild to moderate tricuspid regurgitation with pressure gradient of 113 mm Hg, significant dilation of the right ventricle, and reduced systolic and diastolic function of the right ventricle.

**Take-Home Messages**

• To recognize the importance of RPS and V-P ECMO in the treatment of pulmonary hypertension crises.
• By comparing preoperative and postoperative imaging data, the mechanism by which RPS improves cardiac function is better understood.

## Past Medical History

The infant suffered from cyanosis of the lips after crying for 4 months. Echocardiogram indicated a small patent foramen ovale with no other structurally significant intracardiac anomalies and no other medical diagnosis.

## Investigations

**Figure 1:**
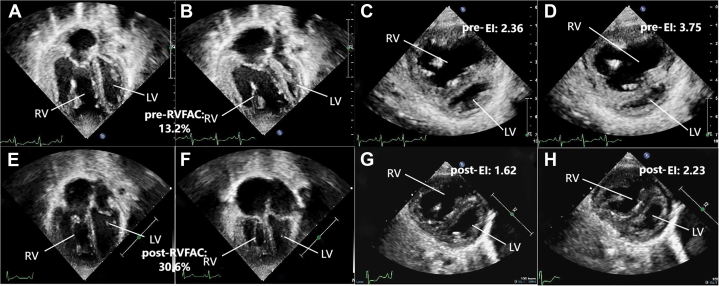
Echocardiographic Assessment of Left and Right Ventricular Function Before and After Surgery (A to D) Preoperative images and (E to H) images 6 months postoperative are shown. (A and B) Apical 4-chamber view showing ventricular size and interventricular septum status at end-diastole and end-systole preoperatively, with RVFAC measured at 13.2%. (C and D) Left ventricular short-axis view showing end-diastolic eccentricity index of the left ventricle (EID) and end-systolic eccentricity index of the left ventricle (EIS) preoperatively, with EID of 2.36 and EIS of 3.75. (E and F) Postoperative apical 4-chamber view showing RVFAC of 30.6%. (G and H) Postoperative left ventricular short-axis view showing EID of 1.62 and EIS of 2.23. EI = eccentricity index; LV = left ventricle; RV = right ventricle; RVFAC = right ventricular fractional area change.

**Figure 2:**
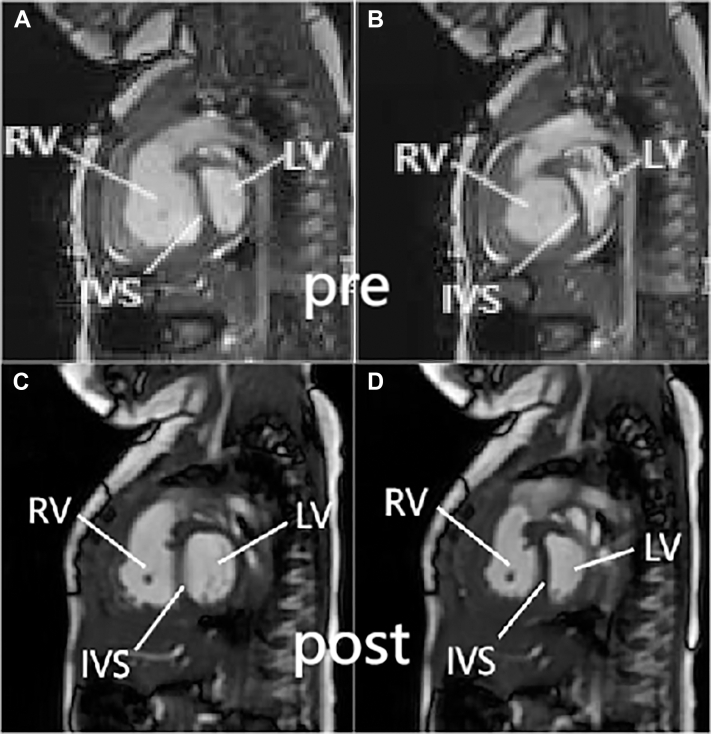
Comparison of Pre- and Postoperative cMRI Images (A and B) Preoperative cardiac magnetic resonance (cMRI) in coronal views demonstrated leftward deviation of the interventricular septum during both systole and diastole, with marked compression and reduction in left ventricular volume during diastole. (C and D) Six-month postoperative contrast-enhanced cMRI exhibited decreased right ventricular volume; the leftward deviation of the interventricular septum during diastole was alleviated, accompanied by significant enlargement of the left ventricle. IVS = interventricular septum. Other abbreviations as in Figure 1.

N-terminal pro–B-type natriuretic peptide (NT-proBNP) levels were >45,000 pg/mL. Whole-exon sequencing revealed a mutation in *TBX4*. Transthoracic echocardiography revealed a tricuspid regurgitation velocity of 5.31 m/s, tricuspid annular plane systolic excursion of 0.97 cm, right ventricular fractional area change (RVFAC) of 13.2%, left ventricular eccentricity index (EI) of 2.36 during diastole and 3.75 during systole, and a pulmonary artery acceleration time of 56 milliseconds (Figure 1). Cardiac magnetic resonance (cMRI) indicated (Figure 2) a significant leftward deviation of the interventricular septum with left ventricular end-systolic volume of 15.66 mL/m^2^, right ventricular end-diastolic volume of 143.32 mL/m^2^, and an impaired right ventricular ejection fraction of 31.19%. The diameter of the descending aorta at the diaphragm level was 6.6 mm. According to the European Pediatric Pulmonary Vascular Disease Network consensus proposed pediatric pulmonary arterial hypertension risk assessment grading scale,^1^ the patient was at high risk based on World Health Organization functional class classification, elevated NT-proBNP levels, and echocardiography/cMRI findings indicating right ventricular dilation and decreased function. As the condition worsened progressively, we considered that right heart catheterization might exacerbate the occurrence of pulmonary hypertension crises, so we did not complete it.

## Management

The infant received preoperative medical therapy consisting of bosentan (12 mg twice a day) and sildenafil (3 mg twice a day) for 1 month before surgery. The dosages were subsequently titrated upward after 15 days to bosentan 16 mg twice a day and sildenafil 6 mg twice a day, and continuous intravenous infusion of epoprostenol for 8 days at a dose of 20 to 60 ng/kg/min. He still suffered from frequent episodes of pulmonary hypertension (PH) crisis manifested as shortness of breath, hypoxia, and tachycardia. Despite the use of mechanical ventilation and inhaled nitric oxide at 20 ppm and norepinephrine at 0.05 μg/kg/min for 1 day, there was no improvement in symptoms, and the patient persistently had a heart rate of 190 beats/min, oxygen saturation of 80%, cerebral and splanchnic oxygen saturation both <45%, and lactate elevated to 7 mmol/L. An emergency reversed Potts shunt (RPS) was performed under midline sternotomy, and a 4-mm anastomosis (60% of the diameter of the descending aorta at the diaphragmatic level) was created between the descending aorta and the origin of the left pulmonary artery using a side-to-side anastomosis under cardiopulmonary bypass. Because of right ventricular swelling and hypoxemia, the patient was unable to be weaned from cardiopulmonary bypass. Two cannulas were inserted separately into the right atrium and the main pulmonary artery, and after connecting to an oxygenator, venopulmonary extracorporeal membrane oxygenation (V-P ECMO) was established. The flow rate was maintained at 100 mL/kg, with conventional mechanical ventilation provided. During V-P ECMO, daily bedside echocardiography was performed to assess right ventricular function. The recovery was slow in the early stage, and the flow rate could not be decreased. One week later, the patient was able to tolerate gradual reduction of the flow rate. V-P ECMO was successfully weaned off and sternal closure surgery was performed after 10 days. In the early period after ECMO decannulation, the difference in oxygen saturation between the upper and lower limbs was about 10%, which decreased to around 5% 1 week later. The infant was extubated from the ventilator after 17 days and transferred out of the intensive care unit 20 days after the surgery. The patient was eventually discharged with dual therapy of bosentan and sildenafil.

## Follow-Up

**Figure 3:**
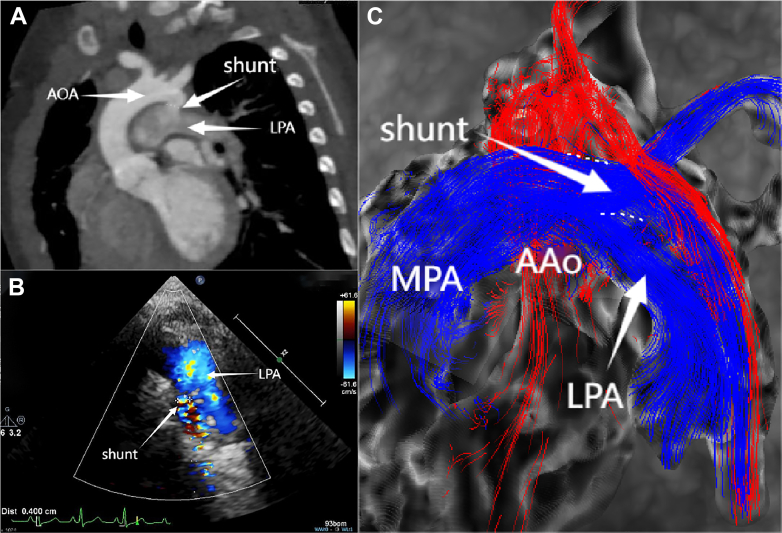
Evaluation of the Size and Direction of the Reversed Potts Shunt Using 3 Imaging Modalities (A) Cardiac computed tomography imaging showing the location and size of the RPS. (B) Echocardiography showing a shunt size of approximately 0.4 cm with bidirectional flow, predominantly right to left (blue flow indicates right-to-left shunting). (C) Cardiac magnetic resonance shows streamline visualization of systemic and pulmonary circulation, with blue indicating pulmonary artery flow and red indicating aortic flow, and pulmonary artery flow is seen entering the descending aorta through the shunt. AAo = ascending aorta; AOA = aortic arch; LPA = left pulmonary artery; MPA = main pulmonary artery; shunt = reversed Potts shunt.

Under the dual medication regimen of oral bosentan and sildenafil, this patient's clinical condition was significantly improved without any PH crisis during the 6-month follow-up. The transcutaneous saturation difference was about 5% between the upper and lower limbs, and NT-proBNP was 286 pg/mL. Echocardiography showed an RPS with a diameter of 0.4 cm, with bidirectional shunting predominantly from right to left. Both cMRI and cardiac computed tomography confirmed the patency and direction of the shunt (Figure 3). The echocardiography also indicated tricuspid regurgitation velocity was 4.9 m/s, tricuspid annular plane systolic excursion was 0.9 cm, RVFAC was 30.6%, EI was 1.62 during diastole and 2.23 during systole, and the pulmonary artery acceleration time was 85 milliseconds (Figure 1). All these parameters were improved to varying degrees compared with preoperatively. cMRI indicated a centered interventricular septum, with improved right ventricular function (right ventricular ejection fraction: 49%) and an enlarged left ventricle (left ventricular end-systolic volume: 25.01 mL/m^2^) (Figure 2).

## Discussion

RPS has been suggested as a bridge to recovery or transplant in severe and refractory PH.^2^ The innovation of this case report lies in the combined application of V-P ECMO and the RPS for treating refractory pulmonary hypertensive crisis episodes in a young infant. Furthermore, imaging examinations confirmed significant postoperative improvements in biventricular size and function compared with preoperative status, thereby explaining the alleviation of the infant's clinical symptoms after surgery.

Based on the estimated pulmonary artery pressure from echocardiography and the descending aorta diameter at the diaphragm level measured by cMRI, an anastomotic diameter of 4 mm was selected. Considering the adjustability of the shunt diameter as the child grows, direct anastomosis was chosen without the use of artificial grafts.

It has been reported that for patients with severe lung disease leading to hypoxemia and right heart failure, but with preserved left heart function, V-P ECMO is an effective supporting approach.^3^ To our knowledge, this could be the first case using V-P ECMO as an auxiliary method to treat children with right heart failure after RPS. V-P ECMO provides hemodynamic support by reducing right ventricular preload and enhancing oxygenation in the pulmonary circulation.^4^ With venous-arterial extracorporeal membrane oxygenation, hypoxemic pulmonary arterial flow may undergo right-to-left shunting retrograde to the descending aorta through the Potts shunt under elevated pulmonary vascular resistance, potentially causing lower body hypoxemia. V-P ECMO elevates oxygenation within the pulmonary artery, preventing this complication. For patients with pulmonary hypertension and preserved left ventricular function, V-P ECMO represents a valuable therapeutic option, providing targeted support while minimizing complications associated with special postoperative pathologic and physiological state. However, for such patients, there is still debate over whether V-P ECMO or venous-arterial extracorporeal membrane oxygenation is superior; more clinical applications and fundamental research is needed in the future to provide stronger evidence.

## Conclusions

For infants with severe PH, RPS is an option for managing sustained and uncontrolled PH crises. V-P ECMO provides excellent supportive effects for right heart failure. Postoperative imaging findings indicate that in the early postoperative period after RPS surgery, the biventricular function of the child showed significant improvement compared with preoperatively.

## Funding Support and Author Disclosures

This work was supported by the National Clinical Key Specialty Construction Project (10000015Z155080000004), Shanghai Research Center for Pediatric Cardiovascular Diseases (2023ZZ02024), the Project of Shanghai Municipal Science and Technology Commission (23Y31900600), Shanghai Key Laboratory of Clinical Molecular Diagnostics for Pediatrics (20dz2260900), the Innovative Research Team of High-Level Local Universities in Shanghai, and the Guizhou Province Children’s Cardiovascular Technological Innovation Leading Talent Workstation (KXJZ[2024]035). The authors have reported that they have no relationships relevant to the contents of this paper to disclose.
